# Successful Nonsurgical Resolution of Descending Necrotizing Mediastinitis: A Case‐Based Illustration of Clinical Management

**DOI:** 10.1155/crdi/7360270

**Published:** 2026-07-27

**Authors:** Feras Al Lami, Abdulfattah Bengheshir, Mohamed Hadi Mohamed Abdelhamid

**Affiliations:** ^1^ Paediatric ICU Department, Tripoli Children’s Hospital, Tripoli, Libya; ^2^ Paediatric Department, Tripoli Children’s Hospital, Tripoli, Libya; ^3^ Department of Cell Biology and Tissue Culture, Libyan Biotechnology Research Center (BTRC), Tripoli, Libya; ^4^ Documentation and Scientific Committees Office, National Center for Disease Control (NCDC), Tripoli, Libya, ncdc.gov.in

**Keywords:** abscess, case report, dental focal infection, DNM, Ludwig’s angina, pleural effusion

## Abstract

**Background:**

Descending necrotizing mediastinitis (DNM) is a relatively uncommon complication of deep neck infections. However, with inappropriate use of antibiotics, steroids, or underlying medical problems such as immunocompromised conditions, this type of infection can become life‐threatening.

**Methods:**

We report a case of Ludwig Angina with DNM, diagnosed at the end of December 2019. The subject was a 17‐year‐old female admitted to the pediatric intensive care unit after two weeks of clinically significant distress and swelling in her neck, tongue, and lips. The predominant underlying oropharyngeal infection originated from the mandibular 3rd molar.

**Results:**

The patient was successfully treated due to early diagnosis and adequate medical intervention. Supportive medications were highly effective, including bronchodilators to assist in breathing and improve lung air entry, intravenous glucocorticoids like dexamethasone for their anti‐inflammatory effect, and a regimen of antibiotics. No surgical intervention was necessary; the only procedures performed were percutaneous drainage of an abscess in the submandibular area and chest tube insertion for pleural effusion.

**Conclusions:**

Early diagnosis and appropriate medical treatment are critical in managing DNM. Supportive medications and minimally invasive procedures can effectively manage the condition, avoiding the need for extensive surgical intervention. This case highlights the importance of vigilant diagnosis and comprehensive medical management in improving patient outcomes.

## 1. Introduction

Descending necrotizing mediastinitis (DNM) is a complication of odontogenic infection due to the spreading of deep neck infections, such as Ludwig’s angina, peritonsillar abscess, or posttraumatic neck abscess. In the first modern series of patients with DNM published in 1938, Pearse reported that 49% of patients died during their treatment [1]. Moreover, this infection is usually self‐limiting and does not spread to other sites, especially with antibiotics, steroids, and nonsteroidal anti‐inflammatory drugs, which can mask the symptoms of such infections. However, if these infections remain untreated, they may rapidly spread down into the mediastinum, pleural cavities, or even to the pericardium [2].

As the infection spreads along deep cervical fascial planes into the mediastinum, widespread cellulitis, necrosis, abscess formation, and sepsis may occur. In addition, delayed diagnoses and inappropriate drainage of the mediastinum are the main causes of the high mortality of those cases (40%–60%) [3–5].

A previous study by Boscolo‐Rizzo et al. reported that deep neck infections are more common in males (51.9%) than in females (48.1%), and there is no set age for this type of infection [6].

In fact, DNM is a severe disease requiring an immediate initiation of multimodal treatment; the all the more expeditious recognition of DNM is crucially important to promptly initiate an appropriate widespread antibiotic treatment to reduce morbidity and mortality [7]. We report on a case of DNM that was successfully diagnosed and treated with appropriate medical approaches. However, the optimal form of the mediastinal drainage model is still controversial and varies widely. These are thoracotomy, median sternotomy, clamshell incision, subxiphoid approach, transcervical approach, video‐assisted thoracoscopic surgery (VATS), mediastinoscopy, and percutaneous catheter drainage [8, 9].

The diagnosis of DNM was suggested by the laboratory findings, clinical examination, and confirmation using computed tomography (CT). The collection of pus from the neck to the mediastinum was the main evidence of DNM. CT scans of the chest provide important information regarding the extent of mediastinal involvement [9].

To the best of our knowledge, this represents the first documented pediatric case of DNM successfully treated without major surgical intervention, relying solely on aggressive medical therapy and targeted drainage. This outcome underscores the potential of nonsurgical management in carefully selected patients and contributes a novel perspective to the evolving treatment paradigm of DNM.

A case report presents a DNM successfully treated due to early diagnosis and adequate medical treatment. Moreover, in our experience, we believe that only through aggressive combined medical management can the effect of DNM be reversed and be highly morbid.

## 2. Case Presentation

**FIGURE 1:**
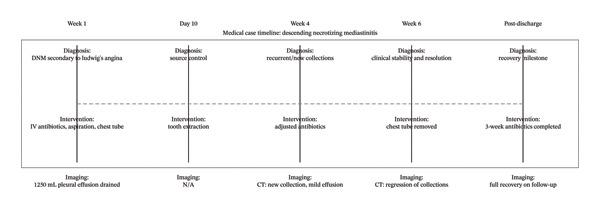
Summarizes the treatment timeline and interventions from admission to discharge.

**FIGURE 2:**
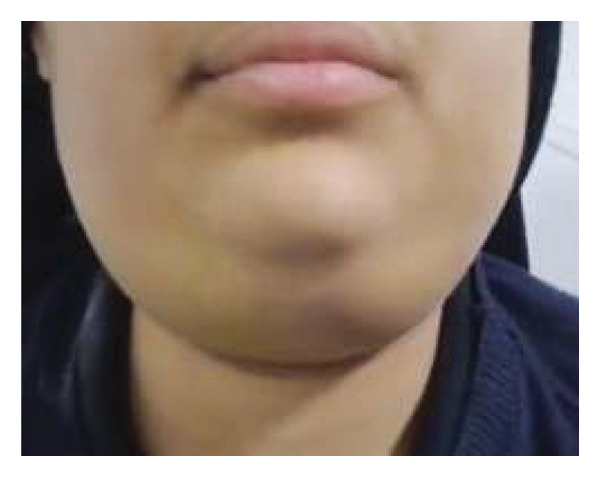
Clinical appearance evidencing extensive submandibular, submental, and sublingual swelling.

We report a case of Ludwig Angina with DNM observed at the end of December 2019 (Figure 1). A healthy 17‐year‐old female was admitted to the pediatric intensive care unit (ICU) at Cartage Clinic, Tripoli, Libya, with a history of swelling in the neck (Figure 2), tongue, and lips for two weeks. A preprint has previously been published [10].

On the initial examination, the patient was leaning forward, had shortness of breath, and complained of dysphagia, aphonia, and regurgitation even with water through the nose. The patient could not lie on the bed and slept in an upright position with orthopnea. She was febrile with a temperature over 38°C, a heart rate of 95–110 b/m, blood pressure of 124/70 mm Hg, respiratory rate of 35–38 b/m, and oxygen saturation of 89%–92%. Examination revealed anterior fullness of the neck, with deep purplish discoloration of her skin.

Significant swelling and induration were present in the submandibular and submental regions extending down towards the base of the neck, and air entry on the right side of the lung decreased markedly. Other physical examinations were unremarkable.

This case report was published as a preprint on ResearchGate[10]. After the family was informed of the treatment options (surgical and medical), they accepted tooth extraction and aspiration of the collected fluid from the submandibular region and multiple other areas, in addition to medical treatment. Moreover, the study was approved by the Ethics Committee (Bioethics Committee at Biotechnology Research Center N^0^: BTC‐BTRC 24_2021). Written informed consent was obtained from the father, the patient’s legally authorized representative. The study was conducted following the Helsinki Declaration.

The diagnosis was DNM arising from Ludwig’s Angina with a dental infection source. Clinical features and presentations supported by the laboratory and radiology exams are requested for diagnostic assistance, such as complete blood count (CBC), renal function test, tuberculin skin test (TST), immunoassay, and tumor markers (Table 1).

**TABLE 1:** Laboratory values throughout hospitalization.

	Reference range	Patient result at time of admission	Patient result during 2nd/W of admission	Patient result in 4th/W of admission (predischarge)
WBC	4–15 *n* × 10^3^ cells/μL	19.1	14.1	7.4
Hemoglobin	9–11 g/dL	8.7	9.6	9.1
Platelet	150–450 × 10^3^/μL	574	436	368
Random serum glucose	70–120 mg/dL	127	85	116
Creatinine	0.6–1.4 mg/dL	0.6	0.7	0.6
Urea	10–50 mg/dL	17	30	29
Na+	135–155 mmol/L	125	133	135.3
*K*+	3.5–5.5 mmol/L	3.63	4	3.59
CL‐	98–107 mmol/L	105	106	100.8
CK‐MB	Up to 25 U/L	17.4		
TROPONIN‐I	Up to 0.30 ng/mL	< 0.01		
CRP	0–0.5 mg/dL	24	96	12
Blood and urine culture	No growth			

We performed ultrasound and CT scans of the neck, chest X‐ray, chest, and abdomen. We collected samples from both submandibular and multiple areas for biochemistry, culture, and sensitivity (C/S) analysis (Tables 2–4).

**TABLE 2:** Laboratory values of fine needle aspiration samples from submandibular swelling which done in 1st week of admission.

*Bacteriology*	
Body fluid differential cell count	
Sample origin	Pus (neck)
Result	
Lymphocytes	20%
Neutrophil	80%
PUS C/S	

*Physical examination*	
Sample origin	Pus (neck)
Color and appearance	Yellowish brown/turbid

*Microscopic examination*	
White blood cells	Moderate
Yeast	Nil

*Culture and identification*	
Culture	No growth after 48 h of incubation

*Gram stain*	
Sample origin	Pus (neck)
Bacteria	No bacteria seen
Yeast	Nil

**TABLE 3:** Laboratory values of pleural fluid samples collected in the 1st week of admission.

*Biochemistry*	
Body fluid LDH	1008 U/L
Body fluid analysis	
Specimen	Pleural fluid
Appearance and color	Slightly turbid/dark yellow
Specific gravity	1.005
Total protein	47.9 g/L
Glucose	46.2 mg/dL
Red blood cells	13,000/mm^3^
White blood cells	500/mm^3^
Lymphocytes	30%
Neutrophil	70%

*Bacteriology*	
Body fluid c/s	
Sample origin	Pleural fluid
Color and appearance	Slightly turbid/dark yellow
Culture	No growth after 48 h of incubation

*Gram stain*	
Sample origin	Pleural fluid
Bacteria	No bacteria seen
Yeast	Nil

**TABLE 4:** Clinical timeline with intervention and treatment management.

Time point	Clinical event/intervention	Antibiotics/procedures	Clinical status	Imaging findings
Week 1 (Admission)	Diagnosis of descending necrotizing mediastinitis (DNM) secondary to Ludwig’s angina	• Broad‐spectrum IV antibiotics: meropenem, vancomycin, metronidazole, ampicillin • Aspiration of 500 mL submandibular pus • Ultrasound‐guided chest tube insertion (1250 mL pleural effusion)	Temp: 38 °C WBC: 19.1 × 10^3^/μL CRP: 96 mg/dL Creatinine, liver enzymes: normal	CT: Large lobulated collections in parapharyngeal and mediastinal spaces; massive right pleural effusion
Day 10	Source control	Extraction of infected mandibular third molar	Temp: 37.8°C WBC: trending down CRP: decreasing	N/A
Week 4	Recurrent/new collections	• Antibiotics changed to imipenem/cilastatin, ceftriaxone, clindamycin, piperacillin/tazobactam	Temp: 37.5°C WBC: 7.4 × 10^3^/μL CRP: 12 mg/dL Renal/liver function: normal	CT: New posterior mediastinal collection; mild recurrent pleural effusion
Week 6	Clinical stability; resolution of collections	Chest tube removed	Temp: 37°C WBC: normalized CRP: near normal	CT: Marked regression of all collections; minimal residual mediastinal fluid
Postdischarge	Recovery milestone	Completion of 3‐week antibiotic course	Afebrile normal labs	Follow‐up confirmed full recovery

The laboratory report of the fine needle aspiration sample of the submandibular swelling, performed on the second day of admission under an ultrasound guide, shows that cytological features are consistent with acute purulent inflammation. In detail, we can say that the samples show mixed inflammatory cells composed of many neutrophils, small lymphocytes, and many foamy macrophages in the background of fibrin, cellular debris, and red blood cells. Multiple scattered mature squamous epithelial cells are seen. No multinucleated giant cells are seen. No atypical or malignant cells were observed in the pleural or submandibular swelling samples (Tables 2–4). Supported by what we say about the misuse of antibiotics, it was a significant risk factor because the patient received 2 weeks of antibiotics, leading to mixed cultures resulting from bacteria.

Furthermore, laboratory data were significant for a 19.1 *n* × 103 cells/μL leukocyte count and a 96 mg/dL C‐reactive protein. The HIV screen was negative.

**FIGURE 3:**
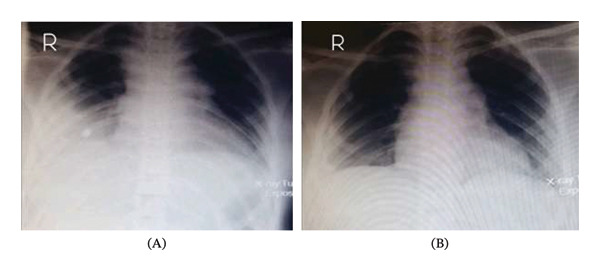
Chest X‐ray showing massive right costophrenic angle obliteration. (A) At the time of admission and (B) before discharge.

A chest X‐ray shows a massive right pleural effusion and blunting of the costophrenic angle (Figure 3). An ultrasound scan of the submandibular area and neck swelling shows right parotid gland hypertrophy with an intracarotid massive abscess, submandibular turbid collection, and multiple inflammatory lymphadenopathies.

The first CT scan of the neck and chest in the 1st week of admission showed a large, well‐defined lobulated collection involving the bilateral. The parapharyngeal and prevertebral cervical spaces, located deep to the platysma muscle, extend into the submandibular regions bilaterally and track inferiorly into the anterior and posterior mediastinum; a right‐sided pleural effusion is also present. spaces and prevertebral cervical spaces, tracking down beneath the platysma muscle, the bilateral submandibular region of the anterior and posterior mediastinum, associated with right‐sided pleural effusion.

**FIGURE 4:**
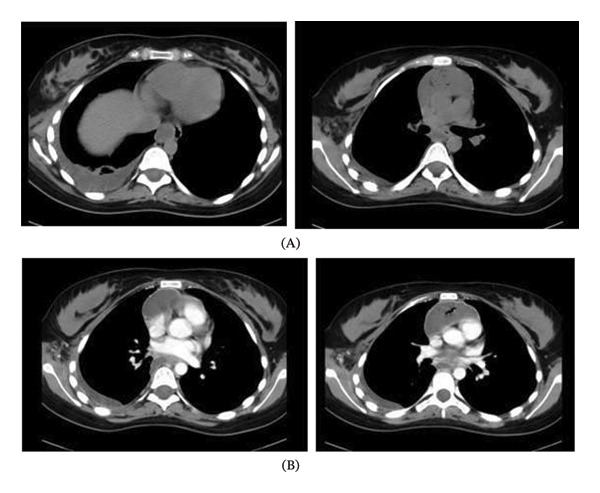
(A) The 2nd computed tomography (CT) scan at 11.01.2020. (B) The 3rd computed tomography (CT) SCAN on 11.2.2020.

On the 2nd CT (S1), the neck and chest SCAN showed contrast at the 4th week of admission (Figure 4A), compared to the previous CT scan of the 1st week. The comparison shows a newly developed small collection socket on the right side of the nasopharynx, 2.0 cm and 1.6 cm posterior to the upper trachea. The unchanged collection is just below the level of the thyroid gland in the anterior lower neck soft tissue.

A newly developed small socket collection is 3.3 cm in axial diameter in the posterior mediastinum and unchanged in the anterior mediastinum, 7.3 × 2.7 cm, with some new postinterventional air inside.

After removal of the right‐sided chest tube with newly developed mild right‐sided pleural effusion, no evidence of suspected pneumothorax, nasopharynx, oropharynx, or larynx appeared normal. However, the bilateral thyroid gland lobes appeared normal. A normal scan of the rest of the bilateral lung parenchyma was performed. The trachea and main bronchi are normal in caliber.

The third CT scan at the 6th week of admission (Figure 4B) (S2), compared with the previous CT scan at the 4th week of admission, shows dramatic regression of the multiple peripherally rim‐enhancing collections previously seen at the upper and lower neck soft tissue, and no more neck soft tissue collection can be detected.

Furthermore, regression of the previously seen collection in the anterior mediastinum and right‐sided pleural effusion shows that there is just a minimal collection seen in the anterior mediastinum, and small, encysted pleural effusions are seen on the right side.

Once DNM was suspected, an experimental broad‐spectrum intravenous antibiotic, including meropenem, vancomycin, metronidazole, and ampicillin, was started as the first line of treatment for 2 weeks.

We believe that administering empirical antibiotics should cover aerobes and anaerobes for possible mixed infection. We then performed another CT scan as an assessment procedure, as a CT of the cervical thorax is the ideal image to evaluate the area of DNM involved. In particular, aspiration of the collection in the submandibular region and right pleural effusion were performed under the complete aseptic technique during the 1st week of admission. The collection from the submandibular area, with 500 mL of pus aspirated, was sent to a laboratory for c/s.

**FIGURE 5:**
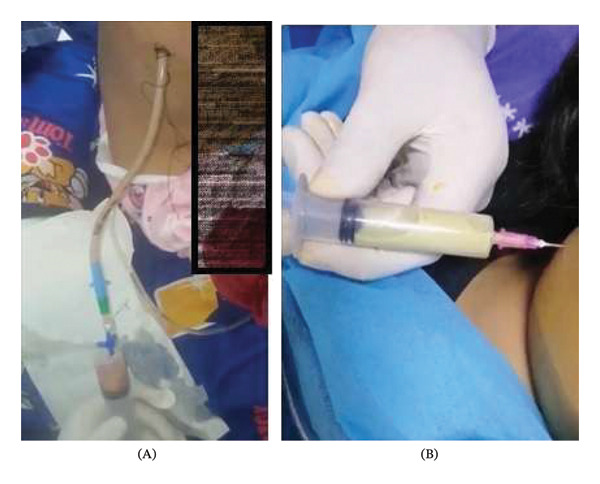
Pictures showing the pus aspirated from the right lower lobe of the chest (A) and right submandibular (B) area.

The pleurocentesis chest tube guided by ultrasound was inserted and drained 1250 mL of pleural fluid and sent to a laboratory for analysis and c/s (Figure 5A,B). Unfortunately, the second CT scan, performed in the 4th week (after aspiration), showed a new collection in the posterior mediastinum and a newly developed mild right‐sided pleural effusion. We changed the antibiotic to imipenem/cilastatin, ceftriaxone, clindamycin, and piperacillin/tazobactam. This decision was made not according to any culture because it was an aseptic collection (Tables 2–4). However, extraction of the 3rd right molar tooth, which had dental caries, was performed, the source of infection, on the 10th day of admission.

The chest and neck CT scan was repeated in the 6th week, showing dramatic regression of the previously seen multiple peripheral rim‐enhancing collections at the upper and lower neck. At the same time, no more neck soft tissue collection can be detected compared to the previous CT scan. The collection was completely resolved, and the patient completed 3 weeks of antibiotics after one month of discharge.

The main clinical symptoms included swelling and pain in the bilateral cervical areas and dysphagia associated with fever. However, for trismus and dyspnea before hospital admission, the patient was receiving oral antibiotics before being admitted to our clinic, which had proven inadequate in preventing the progression of odontogenic or tonsillar infection. Notably, the time between the onset of symptoms and hospital admission was 7 days (Table 4).

In the same vein, diagnostic investigations and radiography after clinical evaluation were performed by CT, which showed swelling and infiltration of the cervical soft tissues, with signs of mediastinal infection (encapsulated fluid collections). Also, postaspiration CT showed regression of the previously seen fluid collections (as supporting video S1 and S2).

After antibiotics and fluid aspiration from the neck and pleural cavities, inflammatory markers changed: CRP decreased from 96 mg/dL in week 2 to 12 mg/dL in week 4, while leukocyte counts dropped from 19.1 × 10^3^ cells/μL in week 1 to 7.4 × 10^3^ cells/μL in week 4. Renal, heart, and liver functions remained stable throughout admission, and no organisms grew in blood or urine cultures.

Moreover, during the therapy and recovery stages, it is crucial to confirm the diagnosis through clinical features, presentations, and supportive laboratory and radiological examinations. Chest tube insertion and neck abscess aspiration were performed within the first days of admission. The specific puncture point for the procedure was in the carotid triangle, which is bordered posteriorly by the anterior border of the Sternocleidomastoid, antero‐inferiorly by the superior belly of the omohyoid muscle, and superiorly by the posterior belly of the digastric muscle.

In fact, the larger and smaller abscesses were drained with antibiotic management. The improvement was clear by ultrasound. After that, the drainage method is ultrasound‐guided needle aspiration, which requires steps. In addition, the next step was to examine the patient to obtain the most prominent area of the abscess, spray the topical anesthetic, and wait several minutes for it to take effect. We used the needle into the mass using the 10‐mL syringe with an 18‐ or 20‐gauge needle and ultrasound. After one week, we adopted the management strategy by changing antibiotics from Ceftriaxone and ampicillin to meropenem and vancomycin. Unfortunately, since the result of all the cultures showed no specific organism, there was no significant improvement on the CT scan for 2 weeks; however, we had to amend the line of antibiotics from meropenem and vancomycin to tazocin and clindamycin, which was a big change in step for the recovery, as the culture was mixed bacteria. During the study, we saw that the most common cultures in this kind of disease resulted in mixed bacteria, as in our case, so we had to start spectrum antibiotics abroad as a combination of resistant Gram‐positive and Gram‐negative bacteria.

The patient was treated at the ICU; however, surgical intervention as an emergency procedure was considered if the condition of the patient deteriorated, as the family refused any surgical intervention if there was an emergency. The medical treatment was supported at some point, as the patient was female and young, and the general condition was good, as she did not need intensive respiratory support. There was no sign of organ failure, and the patient did not have any signs of septic shock.

After needle aspiration and the was adopted of antibiotics, the symptoms were relieved, including orthopnea and sleep disturbance, and the patient did not require tracheotomy or any respiratory support, even by medicine and clinical wiz patient after aspiration of abscess in the neck; also, chest tube drain of the plural effusion there was a big improvement in the patient breathing and condition, and there was no need for surgical intervention as the patient had improved. “Note: In our society, the idea of doing a tracheotomy is a huge event, and that affects the family’s judgment of the condition”. The infection was in the fascia, not by blood, which means inflammation in the anatomical route from the neck to the mediastinum; the patient was very lucky that we started antibiotics and management before any more serious complications started, including septic shock.

Recovery items, including good follow‐up and psychological support of the patient, are very important, especially for her condition; we did not remove or clean any purulent cavity; the follow‐up was for several weeks after discharge, and the results were very successful. In addition, the patient now has a normal life without any complications or discomfort from the disease or our management. Actually, she visited us last month, and she got married.

## 3. Discussion

In this case report, we describe a rare case of DNM due to gram‐negative and gram‐positive multidrug‐resistant bacteria. We obtained the resolution of DNM by combining conventional medical therapy and very strict management, even though there was no recommendation for this strategy in this case, and there was a lack of literature.

Although DNM is predominantly reported in adult populations, pediatric cases have been documented with varying treatment approaches. Ungkanont et al. reported that 37% of deep neck infections in a pediatric cohort were successfully managed with antibiotics alone, without surgical intervention [11]. This finding supports the feasibility of conservative management in selected pediatric patients, particularly when early diagnosis and close monitoring are ensured. Moreover, the literature review by Sumi [8] highlights that among 82 Japanese DNM cases, 15 were managed with percutaneous catheter drainage, suggesting that less invasive strategies may be effective under specific clinical conditions. In the same vein, chest pain, high fever, difficulty breathing, and crackling on palpation are described as symptoms of DNM [12]. In 1836, W. F. V Ludwig described Ludwig’s angina as a rapidly and frequently fatal gangrenous progressive cellulitis and oedema of the soft tissues of the neck and floor of the mouth [13]. In addition, the most common cause of Ludwig’s angina (proximally 46.9%) was dental infection. Interestingly, the submandibular space was the primary site of infection in 84.0% of patients with Ludwig’s angina. However, 16.0% can be involved secondary to an infection of the lateral pharyngeal and parotid spaces. An unsuspecting physician may underestimate an initially localized infection, which could shortly present as airway collapse or descending mediastinitis [4, 6].

Moreover, the inappropriate use of antibiotics, steroids, and nonsteroidal anti‐inflammatory drugs may mask signs of infection and change the clinical presentation, making it more elusive and leading to a slow course of disease, delayed recovery, and the development of complications [14]. However, the combination of a third‐generation cephalosporin such as ceftriaxone with metronidazole or a combination of clindamycin and piperacillin/tazobactam is recommended for empirical antimicrobial therapy in DNM patients [15].

The diagnosis of Ludwig’s angina could be mistaken for a submental abscess, cellulitis of the submaxillary gland, or cervical adenitis. That is why the diagnosis should depend on anatomical and clinical criteria. Anatomically, there must be inflammatory involvement of the sublingual and submaxillary spaces and the tongue, which may be oedematous in some cases, depending on the duration of the disease. On the other hand, clinically, the patient has rounded, tense, brawny, tender swelling that may be unilateral or bilateral. However, the patient has edema, induration, tenderness, and elevation of the floor of the mouth, which leads to difficulty with speech, and in extreme cases, may be unable to talk; difficulty with breathing is the last complaint. It is a sign of chronicity [16].

Appropriate radiological tools help identify the spread between fascial spaces that may not be clinically apparent, and clinical examination alone is not enough, as it may underestimate the presence of disease in 70% of cases [17, 18].

The cervical fascial layers are divided into a superficial and a deep layer. The deep layer consists of three layers (superficial, middle, and deep), generating different deep neck spaces. The dorsal part of the submandibular space offers access to the sublingual compartment and the Parapharyngeal space, which is connected with the retropharyngeal space, and by this anatomical communication route, the spreading of inflammations and infectious processes may reach into the posterior visceral space and cause DNM [19–21]. Pharyngeal spaces communicated with the mediastinum, leading to complications such as pericardial and pleural effusion, as in our case [19, 20].

In the literature between 1970 and 1999, approximately 102 patients with DNM were also identified from 49 published reports. Their mean age ranged from 11 months to 71 years, and most of these patients had mixed aerobic and anaerobic infections, but 4% had the pathogen β‐hemolytic *Streptococcus*. The origin of the infectious process producing DNM was predominantly odontogenic. All of these patients were also treated with intravenous antibiotics [4].

Ludwig’s angina can lead to multiple complications, including DNM, which is a devastating complication, in addition to the even rarer treatment with no major surgical intervention. A differential diagnosis for complications arising from infection in this space includes cranial nerve 9 to 12 palsies, Horner’s syndrome, carotid artery rupture or sheath abscess, and jugular vein suppurative thrombophlebitis (Lemierre’s syndrome). Infection can spread to the retropharyngeal and danger spaces between the posterior border of the pharynx or esophagus and the anterior border of the spine. These spaces communicate with the mediastinum, leading to complications such as pleural or pericardial effusions, as well as DNM [17, 18].

DNM is a very rare complication and diagnosis, especially at this time and after the era of antibiotics. This type of case management is challenging, as physicians are unfamiliar with these uncommon infections. The anatomic communication between these spaces can make a simple, untreated infection become a life‐threatening condition, and the extension beyond the original site of infection sometimes makes it difficult for them to see the main site of infection [2, 18, 20].

Therapeutic needle aspiration of the abscess was considered an alternative to conventional open surgery, and intraoperative findings confirmed the CECT diagnosis in 68%–88% of cases [6].

Five years of published data in Japan, Yuka Sumi, Department of Emergency and Critical Care Medicine, Juntendo University, Urayasu Hospital, Chiba, Japan, presents a comprehensive review of the data regarding DNM in Japan. Twenty‐one patients were treated with video‐assisted thoracic surgical drainage, and 15 cases were treated with percutaneous catheter drainage, whereas the transcervical approach was applied in 25 patients, and thoracotomy was carried out in 21 patients. The overall mortality was 5.6%. Many authors advocated that the most effective management tool is a high degree of clinical suspicion followed by prompt and adequate drainage with intensive care, including hemodynamic and nutritional support, and repeat computer tomographic monitoring [8].

The mortality rate of Ludwig’s angina is less than 10%, but it can increase to more than 30% when complicated by DNM, so we can say that Ludwig’s angina is a potentially life‐threatening condition when it is not treated well [6, 19].

Moreover, management of this condition primarily starts with the surgical choice because the mortality rate increases due to the high risk of septic shock in patients managed late. On the other hand, the appropriate medical management and aspiration of the collected abscess led to a good result, especially a good general condition and the absence of risk factors for septic shock. This position is supported by Ungkanont et al., who successfully treated 37% of DNIs in a series of 117 children exclusively with antibiotics [11].

Notably, the complications of surgery, such as high‐cost procedures, risk of mortality during surgery, recurrence of the drained abscess, risk of anesthetic‐related nausea and vomiting, wound infection, scars, bad cosmetic appearance, and impact of surgery on psychiatric patients, can all be avoided by appropriate medical treatment [22, 23]. Even if nonsurgical treatment is tempting, more data are needed before surgical intervention is considered.

## 4. Conclusion

DNM is an uncommon condition with an often‐nonspecific clinical presentation. To date, medical treatment does not appear to increase complications of deep neck infections, nor does it prolong hospitalization or associate with increased morbidity or mortality. Based on our experience, the case of DNM was successfully treated due to early diagnosis and appropriate medical intervention. Furthermore, we believe that only through aggressive combined medical management—using antibiotics targeting both aerobic and anaerobic organisms—and drainage at all pus collection sites can the highly morbid effects of DNM be effectively reversed.

## Author Contributions

Manuscript writing: All authors.

## Funding

This research did not receive any specific grant from funding agencies in the public, commercial, or not‐for‐profit sectors.

## Disclosure

All authors approved the final version of the manuscript. The manuscript was previously published as a preprint as per the following link: https://www.researchsquare.com/article/rs-1273820/v1 [24].

## Conflicts of Interest

The authors declare no conflicts of interest.

## Supporting Information

Additional supporting information can be found online in the Supporting Information section.

## Supporting information


**Supporting Information** Video S1 and S2: The 2nd CT, 3rd and last CT: The longitudinal CT imaging documented the progression and eventual resolution of the descending necrotizing mediastinitis as follows: the initial scan revealed extensive multiloculated collections in the bilateral parapharyngeal and perivertebral spaces tracking beneath the platysma into the submandibular regions, with involvement of both the anterior and posterior mediastinum and a right‐sided pleural effusion. By the second scan in the first week, while the anterior mediastinal and lower neck collections remained stable, new localized collections developed in the right nasopharynx (2 cm times 1.6 cm) and the posterior mediastinum (3 cm times 3 cm), alongside postinterventional air pockets. Finally, the fourth‐week scan demonstrated dramatic clinical regression of all previously noted peripheral rim‐enhancing collections, with no detectable soft‐tissue collections remaining in the neck or mediastinum.
